# Malaria hotspots and risk factors among children under-five years of age across eight West African countries: A geospatial analysis of DHS data

**DOI:** 10.5281/zenodo.17777419

**Published:** 2025-12-01

**Authors:** Edmond Sacla Aidé, Adama Kazienga, Oyelola Adegboye, Paul Sondo, Halidou Tinto

**Affiliations:** 1 Laboratoire de Biomathématiques et d’Estimations Forestières, University of Abomey-Calavi, Benin.; 2 Institut de Recherche en Sciences de la Santé/ Clinical Research Unit of Nanoro (IRSS-URCN), Burkina Faso.; 3 Menzies School of Health Research, Charles Darwin University, Darwin, NT, Australia.

## Abstract

**Background:**

Malaria remains a significant public health challenge in sub-Saharan Africa, disproportionately affecting children under five years of age. Understanding the spatial distribution of malaria and its associated risk factors is essential for implementing effective, targeted control strategies. In this study, we investigated spatial variation and key determinants of malaria prevalence among children under five in eight West African countries.

**Methods:**

The study used the most recent Demographic and Health Surveys Data from eight West African countries where malaria infection status was determined by microscopy. Generalised Linear Mixed Models were first used to explore associations between malaria infection and sociodemographic predictors, accounting for survey design. These models were extended into Generalised Linear Geostatistical Models to incorporate spatial random effects. Malaria prevalence was predicted at a 10 x 10 km resolution, and exceedance probability maps were generated to identify high-burden areas with prevalence exceeding 30%. Model validation was done using empirical variograms, PIT histograms, and residual spatial analyses.

**Results:**

The use of insecticide-treated mosquito nets was significantly associated with reduced odds of malaria infection in four of the eight countries included in the study, while younger child age (<2 years) was consistently associated with lower risk across all countries. In addition, marked spatial heterogeneity in malaria prevalence was observed, with high predicted prevalence in Benin and Cote d'Ivoire and lower prevalence in Ghana and Liberia.

**Conclusion:**

This study highlights the importance of geospatial approaches for understanding malaria transmission dynamics in order to tailor malaria control measures to local context. The findings underscore the need to strengthen the effective use of insecticide-treated nets and community-level vector control, while improving spatial surveillance and data integration to support context-specific malaria interventions.

## Introduction

Malaria remains a major public health threat, particularly in sub-Saharan Africa, where children under five are disproportionately affected. In 2023, malaria caused approximately 597,000 deaths globally, with children under five accounting for 76% of these deaths [1,2]. The WHO African Region carries the highest burden, contributing 94% of cases and 95% of malaria-related deaths worldwide [1,2]. Within West Africa, malaria continues to cause substantial morbidity and mortality despite widespread control efforts [3,4]. Factors such as climate change, increasing insecticide and drug resistance, and disruptions from the COVID-19 pandemic have contributed to the persistence of malaria in the region [3,4].

Malaria burden varies significantly across West African countries due to differences in socioeconomics characteristics, ecological conditions, health system capacity, and intervention coverage. For instance, in 2023, the highest malaria incidence rates were reported in Benin (363 per 1,000), Burkina Faso (353 per 1,000), Mali (346 per 1,000), and Guinea (307 per 1,000), whereas Senegal recorded the lowest (66 per 1,000) [5]. Understanding such national variations is essential for implementing targeted control strategies.

Malaria control relies on a combination of interventions, including insecticide-treated nets (ITNs), indoor residual spraying (IRS), chemoprophylaxis, preventive chemotherapies, vaccination, and early diagnosis with prompt treatment [1,6]. However, in many West African countries, weak health information systems limit routine surveillance and evaluation, underscoring the need for reliable alternative data sources. Population-based surveys, such as the Demographic and Health Surveys (DHS), provide standardised national and subnational health indicators, including malaria infection confirmed by microscopy, and serve as valuable tools for evidence-based planning [7,8].

Previous studies in sub-Saharan Africa have identified several individual- and household-level factors associated with malaria infection among children under five. These include child age, sex, maternal education, socioeconomic status, residence, and ITN usage [9,10]. Region-specific studies in West Africa confirm these associations: in Benin, high household density, low socioeconomic status, and poor bednet conditions increased infection risk [11]; in Burkina Faso, household wealth, maternal education, and ITN use were significant determinants [12,13]; in Cote d'Ivoire, infection was associated with child age, sex, and household socioeconomic status [14]; and in Ghana, residential area, child age, and mosquito net ownership influenced risk [15,16]. Multi-country analyses further highlight the consistent role of these determinants in shaping malaria burden [17].

Traditionally, these factors have been studied using logistic regression, multilevel, or mixed-effects models [14,17,,18]. While informative, such approaches often ignore spatial dependence, limiting their ability to identify high-risk areas and guide geographically targeted interventions. Spatial correlation is particularly important in regions with heterogeneous ecological and sociodemographic conditions, where malaria transmission dynamics vary across small geographic scales.

Geospatial modelling approaches, particularly model-based geostatistics, address this limitation by incorporating spatial correlation, environmental covariates, and uncertainty to generate high-resolution risk maps [19,20]. These models not only reveal spatial heterogeneity but also facilitate subnational evaluation of intervention impact, supporting more efficient and equitable malaria control strategies [21]. Previous applications of geostatistical models in malaria research have successfully identified high-risk areas and associations with environmental, demographic, and socioeconomic factors [22-24].

Despite these advances, few multi-country geostatistical studies using microscopy-confirmed DHS data have been conducted in West Africa. This gap limits understanding of spatial heterogeneity in malaria prevalence among children under five and constrains the targeting of interventions in the region. This study addressed this gap by applying model-based geostatistics to identify high-risk areas and determinants of malaria infection across eight West African countries.

The objectives of this study were to: (i) estimate malaria prevalence among children under five years of age; (ii) identify individual- and household-level determinants of infection; and (iii) map spatial heterogeneity and detect high-risk areas to guide targeted malaria control efforts.

## Methodology

### Study design and population

The latest Demographic and Health Survey (DHS) data for thirteen West African countries was used. These DHS surveys employed a two-stage stratified cluster sampling design to ensure national representativeness. In the first stage, enumeration areas (clusters) were randomly selected from national census sampling frames. In the second stage, households were systematically sampled from the selected clusters. Household and individual-level weights were applied to adjust for survey design effects and ensure the sample's representativeness at national and sub-national levels. Also, countries were divided into strata based on urban and rural areas, with further stratification.

The study population was children under five living in sampled households in the DHS survey of the selected countries. This study initially considered the most recent Demographic and Health Surveys (DHS) data from thirteen West African countries: Benin, Burkina Faso, Cote d'Ivoire, Ghana, Gambia, Guinea, Liberia, Sierra Leone, Senegal, Togo, Niger, Nigeria, and Mali. However, only eight countries were retained in the final analysis (Benin, Burkina Faso, Cote d'Ivoire, Ghana, Guinea, Liberia, Nigeria, and Togo). The selection was based on the availability of malaria infection status determined by microscopy. Using microscopybased malaria infection data provides a standardised and highly specific measure of *Plasmodium falciparum* infection. This approach is particularly important in multi-country analyses, where consistency in diagnostic methods enhances the comparability and validity of spatial and statistical modelling results. The five excluded countries (Gambia, Sierra Leone, Senegal, Niger, and Mali) were omitted because microscopy results were either unavailable, incomplete, or based solely on RDTs, preventing harmonised data analysis across all countries (Table 1).

**Table 1:** Reasons for omitting countries in the analysis.

Country	DHS Year	Malaria Infection Test Type	Reason for Exclusion
Gambia	2019	RDT only	Microscopy data unavailable
Sierra Leone	2019	Not available	Not available
Senegal	2023	Not available	Not available
Niger	2021	RDT only	Microscopy data unavailable
Mali	2021	RDT only	Microscopy data unavailable

The outcome variable was the microscopy-confirmed *Plasmodium falciparum* infection status among children aged 6-59 months living in sampled households. For each survey cluster *i*, the number of malaria-positive children (*y^_i_^*) out of the total examined (*n_i_*) was recorded, with *N* denoting the total number of clusters. Thus, malaria prevalence at each cluster location (*x_i_*) was defined as *yi/n_i_.*

The selection of covariates was informed by previous studies on malaria risk factors among children under five and by the availability of comparable variables across all eight DHS datasets [19,33]. Only individual- and household-level variables consistently measured in the surveys were included, namely: child age, child sex, maternal education, maternal age, household head sex, and bednet ownership or use. These variables have been widely reported as major sociodemographic determinants of malaria risk. Other relevant factors such as housing characteristics, environmental, and climatic covariates were not incorporated due to their absence or inconsistency in the DHS datasets. Future analyses integrating external geospatial datasets could help capture these ecological influences on malaria transmission dynamics more comprehensively.

**Figure 1:**
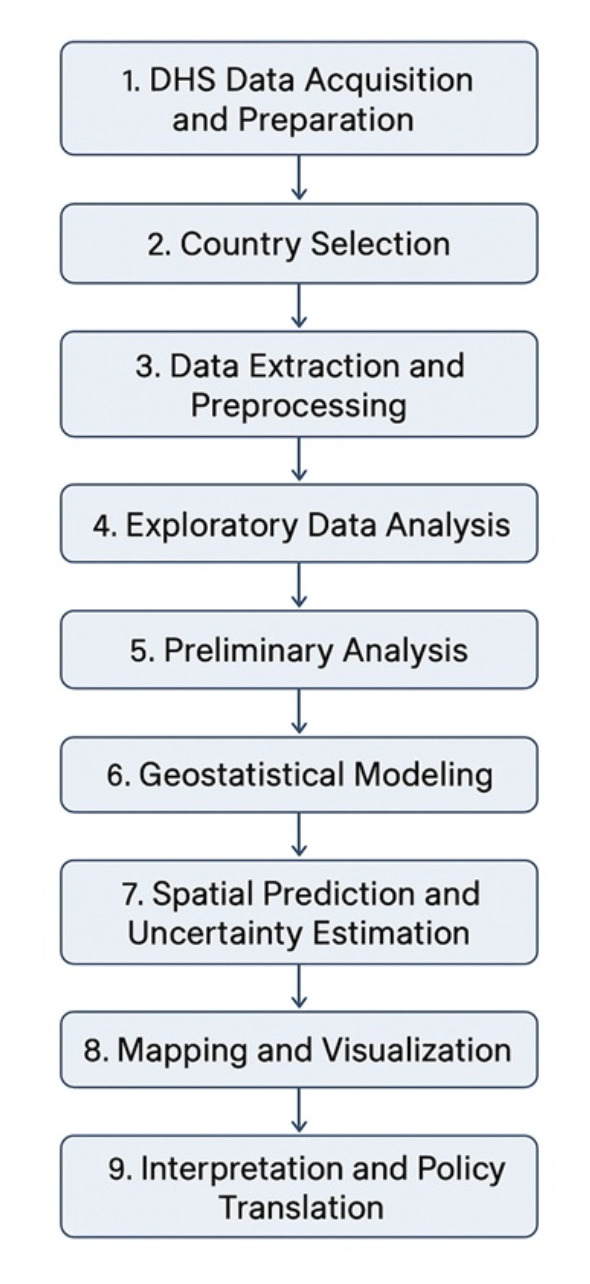
Data processing and workflow analysis for malaria risk mapping across eight West African countries. The flowchart summarises the sequential steps undertaken in this study. The workflow highlights the integration of nationally representative microscopy-based DHS data with spatial modelling to identify malaria hotspots and support evidence-based targeting by national malaria control programmes.

In addition to these sociodemographic data, geographical coordinates were recorded for each survey cluster, allowing for spatial analysis of malaria prevalence and associated risk factors. To maintain confidentiality, DHS applies a displacement procedure to the cluster coordinates, ensuring anonymity while preserving spatial accuracy for large-scale epidemiological studies. An overview of the data processing and analytical workflow, from DHS data acquisition to geostatistical modelling and visualisation, is presented in Figure 1.

### Geostatistical model

A Generalised Linear Mixed Model (GLMM) using the package survey [4] was first employed to investigate the factors associated with malaria infection in children under five. The objective was to determine which covariates, within a given set, showed a statistically significant association with malaria infection. This model accounted for both fixed and random effects to ensure robust estimates in the presence of hierarchical survey data. This survey design included stratification, clustering, and sampling weights to ensure unbiased population-level inference.

Let *Y_i_* denote the number of microscopy-confirmed malaria-positive children out of *n_i_* examined at location *X_i_,* where i=1,...,*N*. The malaria prevalence at location *x_i_* is defined as *p(x_i_)=Yi/n_i_*. The number of positive cases is assumed to follow a binomial distribution:

Yi~Binomialni,pxi

The linear predictor is specified as:

logitpxi=dxiTβ+Sxi+Zi

where *d(xß)* is the vector of covariates at location *X_i_, ß* represents fixed-effect coefficients, *S*(*x_i_*) is a spatially structured Gaussian process capturing spatial correlation, and *Z_i_* is an independent random effect (nugget effect) accounting for small-scale variability. The spatial process *S*(*x_i_*) is assumed to follow a zero-mean stationary Gaussian process with covariance function:

CovSxi, Sxi=σ2exp−∥xi−xj∥/φ

where *σ2* is the spatial variance and *φ* is the range parameter that controls the rate of spatial correlation decay. Model parameters *ß*, *σ*2, *φ* and *τ2* were estimated using the Monte Carlo Maximum Likelihood (MCML) method as implemented in the PrevMap R package [35,36]. Unlike Bayesian approaches, MCML does not require prior specification for these parameters, treating them as unknown constants estimated directly from the data.

Predictions were performed on a regular grid with a spatial resolution of 10 x 10 km to generate spatially continuous malaria prevalence estimates. This resolution was chosen as a pragmatic balance between spatial detail and the positional uncertainty of DHS cluster coordinates (DHS implements random displacement of cluster locations urban up to 2 km, rural up to 5 km, with a small proportion displaced up to 10 km), which limits the meaningful spatial precision of survey-based prevalence estimates. A 10-km pixel therefore avoids over-interpreting fine-scale patterns that the input data cannot support, while remaining sufficiently fine to identify subnational hotspots relevant for programmatic decision-making. In addition, the 10-km grid offered a computationally tractable number of prediction locations for Monte Carlo maximumlikelihood estimation and yielded a spatial support compatible with commonly used environmental covariates.

This predictive prevalence was obtained as the posterior mean of *p*(*x*) at each grid location, incorporating both spatially structured and non-structured effects. To this end, exceedance probability (EP) represents the probability that malaria prevalence exceeds a predefined threshold to identify high-risk areas. This threshold was fixed at 30%, indicating that areas where the predicted malaria prevalence had a probability of exceeding 30% were classified as high-risk regions. This prevalence threshold was used to delineate areas with a high malaria burden, warranting intensive and sustained vector control interventions, as supported by previous studies [25,26]. These predicted prevalence and exceedance probability maps were generated to highlight regions with a high burden of malaria visually. These maps provide valuable insights for targeted malaria control interventions, allowing public health officials to allocate resources more effectively and implement strategic prevention measures. To ensure comparability across countries, a consistent legend scale was applied to all maps.

**Figure 2:**
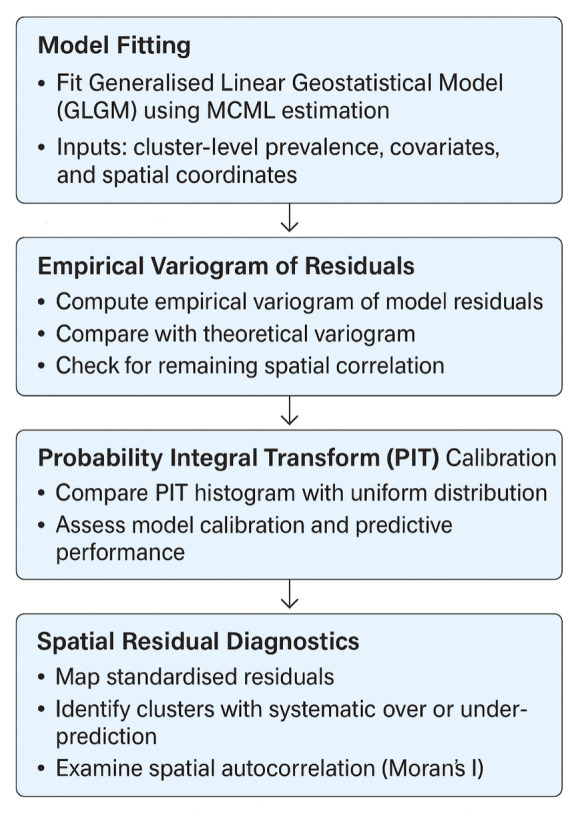
Schematic overview of the model validation workflow. The diagram illustrates key validation steps used to assess the performance of the geostatistical model: (1) computation of empirical variograms of residuals; (2) PIT-based model calibration check; (3) examination of standardised residuals for remaining spatial structure; and (4) interpretation of diagnostics for model adequacy.

The performance of the fitted geostatistical models was evaluated using three complementary diagnostics: (i) the empirical variogram of residuals to assess whether residual spatial correlation was adequately captured; (ii) the probability integral transform (PIT), which compares predicted probabilities with observed outcomes to evaluate model calibration; and (iii) the spatial distribution of standardised residuals to identify any remaining spatial patterns unexplained by the model. A schematic overview of the validation workflow is provided in Figure 2 for clarity.

The empirical variogram of these residuals was then computed and compared against a 95% confidence envelope derived from permutation tests. If residual spatial correlation remained, it suggested that important spatially structured factors influencing malaria prevalence had not been accounted for, warranting further model refinement.

### Software

Data management and variable recording were conducted using Stata 17 (StataCorp, 2021). All statistical analyses were performed in R version 4.3.1 (R Core Team, 2023), with statistical significance set at p < 0.05. Generalised Linear Mixed Models (GLMMs), accounting for complex survey design, were implemented using the survey package [4]. Parameter estimation, spatial prediction, and model validation for the geostatistical models were conducted using the PrevMap package [27,28].

### Ethical considerations

This study utilised publicly available secondary data from the Demographic and Health Surveys (DHS) Programme. The DHS surveys follow rigorous ethical protocols approved by the ICF Institutional Review Board and by the national ethics committees of each participating country. Informed consent was obtained from all participants prior to data collection. The datasets are fully anonymised and contain no identifiable information on survey respondents. Prior to accessing the data, the corresponding author registered on the DHS Program website and was granted permission to download and use the datasets for this research. Data access and ethical guidelines are publicly available at https://dhsprogram.com/data/.

## Results

### Malaria infection risk factors for children < 5 yrs

Out of the 13 countries surveyed, only eight had complete malaria infection status based on microscopy and were therefore included in the analysis. Table 2 presents the estimated associations between selected demographic and household-level factors and malaria infection among children under five across these eight countries in West Africa. Child sex, maternal education, and the mother's age were not significantly associated with malaria infection in any of the countries included. Similarly, sex of household head showed no association with malaria risk, except in Cote d’Ivoire, where children living in male-headed households had significantly higher odds of malaria infection (OR = 1.30, p = 0.029). In addition, the use of insecticide-treated bednets was significantly associated with reduced malaria risk in four of the eight countries (Burkina-Faso, Benin, Ivory Coast, and Ghana). For instance, children who slept under treated nets (such as long-lasting insecticidal nets) had 28% lower odds of malaria infection (OR = 0.72, p < 0.001) compared to those who did not use a net. In contrast, the age of the child was significantly associated with malaria infection across all eight countries. For example, children under the age of 2 years had 40 to 63% lower odds of malaria infection compared to those above 2 years old.

**Table 2:** Factors associated with the prevalence of malaria in children < 5 yrs of age in eight West African countries (CI = 95% confidence interval; HH = Head of Household).

Covariate	Category	Burkina Faso		Benin		Cote d’Ivoire		Ghana	
		Odds Ratio (CI)	P value	Odds Ratio (CI)	P value	Odds Ratio (CI)	P values	Odd Ratios (CI)	P value
Child sex	Ref: Female	-	-	-	-	-	-	-	-
	Male	1.0151 (0.861,1.219)	0.865	1.053 (0.946,1.179)	0.357	1.101 (0.943,1.283)	0.218	1.052 (0.796,1.369)	0.708
HH sex	Ref: Female	-	-	-	-	-	-	-	-
	Male	1 -246 (0.916,1.721)	0.171	0.955 (0.798,1.145)	0.613	1-296(1.028,1.650)	0.029	0.901 (0.658,1.237)	0.513
Use of net	Ref: No	-	-	-	-	-	-	-	-
	Yes	0.719 (0.598,0.879)	<0.001	0.711 (0.615,0.867)	<0.001	1.566(1.345,1.919)	<0.001	1-455 (1.140,1.966)	0.006
Child age	Ref: > 2 yrs	-	-	-	-	-	-	-	-
	< 2 years	0.607 (0.506,0.734)	<0.001	0.625 (0.554,0.705)	<0.001	0.574 (0.488,0.673)	<0.001	0.538 (0.405, 0.714)	<0.001
Mother education	Ref: None	-	-	-	-	-	-	-	-
	Primary	2.311 (0.819,16.486)	0.192	1.932 (0.146,18.854)	0.596	0.967 (0.512, 2.005)	0.606	1.282 (0.284,4.065)	0.713
	Secondary	3.382 (0.937,5.248)	0.093	(0.172,19.162)	0.456	1.673 (0.390,1.924)	0.583	1.467 (1.183,2.947)	0.372
	Ref: <24	-	-	-	-	-	-	-	-
Mother age (yrs)	24-35	1.083 (0.738,2.562)	0.472	0.967 (0.437,3.837)	0.645	1.102 (0.737,2.837)	0.558	0.945 (0.364,1.974)	0.632
	35-50	1.015 (0.678,2.893)	0.772	1.031 (0.726,2.274)	0.583	0.991 (0.426,1.974)	0.842	1.028 (0.473,2.037)	0.719
	>50	0.958 (0.627,1.936)	0.669	1.068 (0.936,4.437)	0.396	1.120 (0.836,4.243)	0.378	1.056 (0.374,0.743)	0.443
									
Covariate	Category	**Guinea**		**Liberia**		**Nigeria**		**Togo**	
		Odds Ratio (CI)	P Value	Odds Ratio (CI)	P Value	Odds Ratio (CI)	P Value	Odds Ratio (CI)	P Value
Child sex	Ref: Female	-	-	-	-	-	-	-	-
	Male	1.075 (0.897,1.276)	0.418	1.143(0.883,1.524)	0.322	1.003(0.897,1.132)	0.958	1.076(0.888,1.292)	0.430
HH sex	Ref: Female	-	-	-	-	-	-	-	-
	Male	1.430 (0.966,2.189)	0.082	1.265 (0.837,2.017)	0.284	1.151 (0.915,1.544)	0.283	1.178 (0.917,1.572)	0.224
Use of net	Ref: No	-	-	-	-	-	-	-	-
	Yes	1.019 (0.823,1.300)	0.868	0.744 (0.542,1.107)	0.100	0.993 (0.872,1.164)	0.927	1.309 (0.997, 1.818)	0.073
Child age	Ref: > 2 yrs	-	-	-	-	-	-	-	-
	<2 years	0.601 (0.480,0.749)	<0.001	0.371 (0.254,0.541)	<0.001	0.508 (0.445,0.580)	<0.001	0.511 (0.424,0.614)	<0.001
	Ref: None	-	-	-	-	-	-	-	-
Mother education	Primary	2.291 (1.819,6.486)	- 0.297	3.132 (1.573,5.738)	- 0.496	0.495 (0.172,0.926)	- 0.108	1.578 (1.266, 2.889)	- 0.513
	Secondary	2.927 (1.937,7.248)	0.193	2.487 (1.282,4.833)	0.357	1.933 (1.183, 3.936)	0.333	1.726 (1.445,2.219)	0.179
	Ref: <24	-	-	-	-	-	-	-	-
Mother age (yrs)	24-35	1.045 (0.737,4.837)	0.538	1.061 (0.837,2.337)	0.487	0.986 (0.826,3.648)	0.743	1.072 (0.673,2.037)	0.621
	35-50	0.979 (0.626,5.274)	0.682	0.992 (0.526,1.574)	0.916	1.058 (0.836,3.362)	0.392	0.988 (0.564,1.774)	0.832
	>50	1.022 (0.536,6.437)	0.794	0.975 (0.626,1.474)	0.703	1.034 (0.737,2.682)	0.614	1.009 (0.836,43437)	0.925

### National prevalence of malaria infection

National-level malaria prevalence based on microscopy was estimated for each of the eight included countries, with corresponding 95% confidence intervals as shown in Table 3. Overall, there was significant geographical variation in malaria prevalence across the eight countries, reflecting differences in transmission intensity, environmental conditions, and control efforts. For instance, the highest prevalence was recorded in Benin (39.73%, 95% CI: 38.49%-41.00%), followed by Togo (29.83%, 95% CI: 28.25%-31.46%), whereas Ghana had the lowest prevalence (9.96%, 95% CI: 9.10%-10.89%).

**Table 3:** National prevalence of malaria according to DHS surveys (CI = 95% confidence interval).

Country	DHS Survey	Prevalence (%)	95% CI
Burkina Faso	2021	13.15	12.29 -14.06
Benin	2017	39.73	38.49 - 41.00
Cote d'Ivoire	2021	29.14	27.85 - 30.47
Ghana	2022	9.96	9.10 -10.89
Guinea	2021	16.45	15.32 -17.65
Liberia	2022	11.84	10.68 -13.09
Nigeria	2021	21.25	20.47 - 22.04
Togo	2017	29.83	28.25 - 31.46

### Malaria prevalence at sampled cluster locations

**Figure 3:**
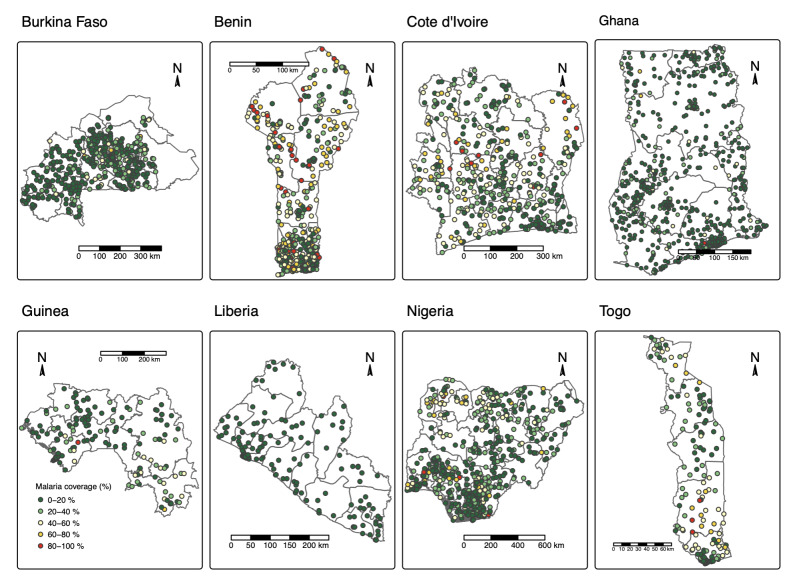
Observed malaria prevalence at DHS cluster locations across eight West African countries. Each point represents the microscopy-confirmed malaria prevalence among children under five at the DHS cluster level. Source: Demographic and Health Surveys (DHS), 2017-2022; authors’ analysis.

The prevalence at sampled cluster location was estimated among children under five years across the eight countries, as shown in Figure 3. The prevalence at the sampled location exhibits substantial spatial heterogeneity. In countries such as Burkina Faso, Ghana, and Guinea, most sampled clusters exhibited low to moderate malaria prevalence levels, with prevalence frequently below the 30% threshold. In contrast, Benin, Cote d'Ivoire, Nigeria, and Togo present wider variations, with several clusters showing higher malaria prevalence (above 40%), particularly in southern Benin and parts of Nigeria and Togo. Meanwhile, Liberia exhibits relatively moderate malaria prevalence across most of its regions, with limited pockets of very high prevalence.

### Model validation

**Figure 4:**
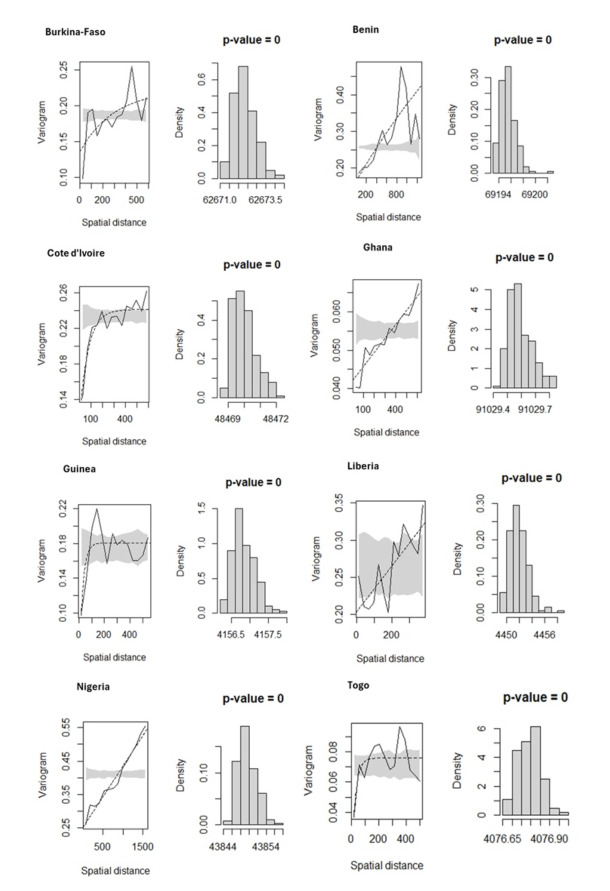
Test for spatial autocorrelation of malaria prevalence among children under five across eight West African countries. The figure shows the results of spatial autocorrelation analysis using Moran's I statistic applied to cluster-level malaria prevalence derived from DHS data. A positive and statistically significant Moran's I indicates spatial clustering of malaria risk across the study area. Source: Demographic and Health Surveys (DHS), 2017-2022; authors' analysis.

**Figure 5:**
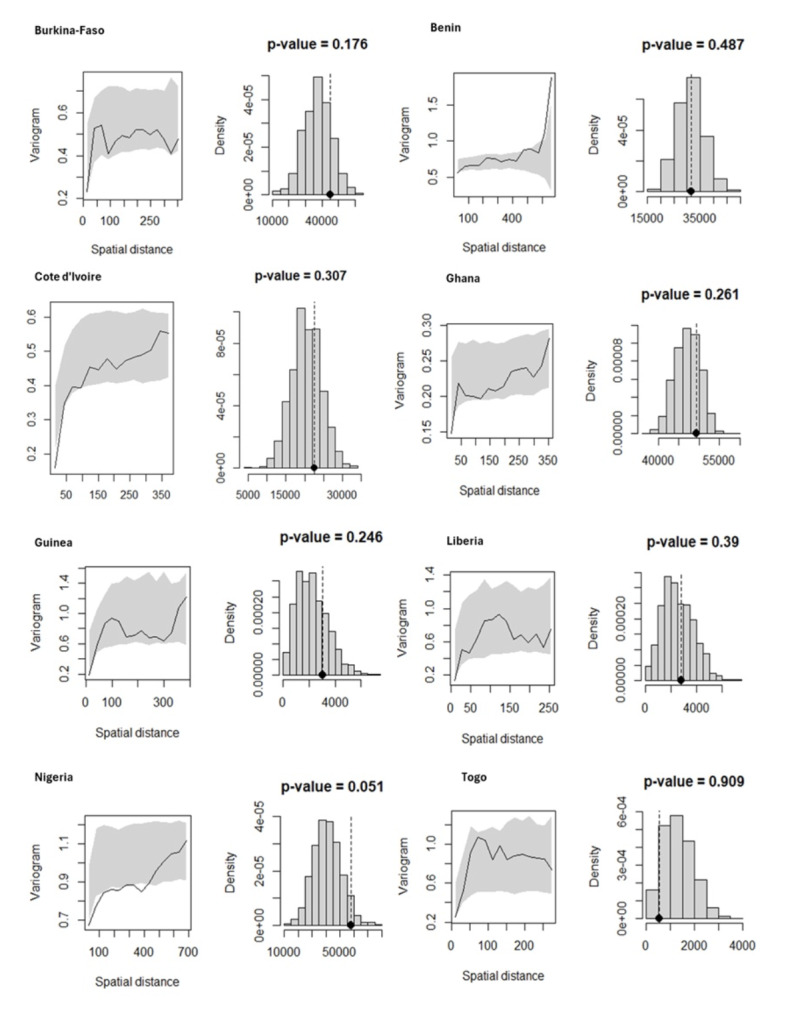
Model validation of Bayesian geostatistical predictions of malaria prevalence. Posterior predictive checks comparing observed and predicted malaria prevalence at DHS cluster locations. The figure demonstrates the model's goodness of fit and reliability of spatial predictions, indicating that the Bayesian geostatistical model accurately captures observed spatial patterns. Source: Demographic and Health Surveys (DHS), 2017-2022; authors' analysis.

To investigate malaria risk factors when accounting for spatially structured random effects, a GLGM was fitted, and model validity was assessed through spatial diagnostics for malaria infection across the eight countries. The results provided insufficient evidence to reject the presence of spatial correlation, indicating that malaria prevalence was spatially dependent across included countries (Figure 4). Additionally, model diagnostics revealed no evidence against the assumed exponential correlation structure as shown in Figure 5, supporting the overall validity of the fitted model across all countries.

### Predicted malaria prevalence across West African countries

**Figure 6:**
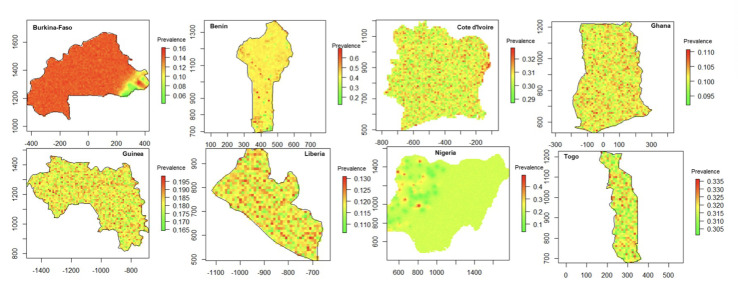
Predicted malaria prevalence across eight West African countries based on Bayesian geostatistical modelling. Predicted values are shown at a 10 x 10 km spatial resolution. Source: DHS, 2017-2022; authors'

Figure 6 presents model-based predictions of malaria prevalence maps at a 10 km x 10 km resolution, offering a fine-resolution depiction of malaria burden across the included countries. Overall, Figure 6 suggests that high malaria prevalence areas are localised rather than widespread, except for Benin and parts of Cote d'Ivoire, where broader regions with elevated prevalence are observed. In addition, most mapped areas display low predicted malaria prevalence, typically below 20% (Burkina Faso, Guinea, Ghana, and Liberia). In contrast, in Benin, malaria prevalence is predicted to be moderately high in the southern part of the country, while the north exhibits lower levels. Similarly, Cote d'Ivoire shows notable spatial variation, with elevated prevalence predicted in central and eastern regions, while Nigeria's map highlights a wide range of predicted values, although large parts of the country show moderate prevalence levels. Finally, Togo exhibits a relatively homogeneous malaria burden, with a large proportion of the country presenting moderate prevalence (around 30%).

### Predicted probability of malaria prevalence exceeding the 30% threshold

**Figure 7:**
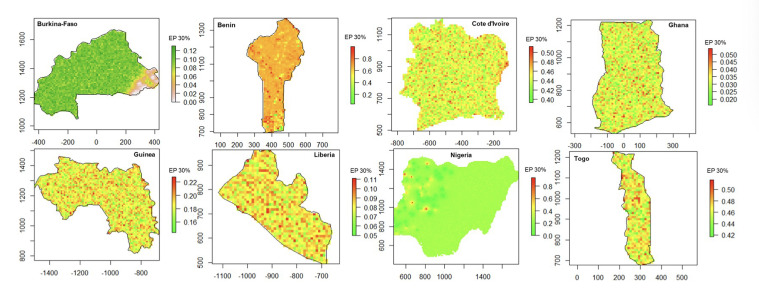
Probability of malaria prevalence exceeding the 30% threshold across eight West African countries. Areas with higher probabilities (in red) indicate persistent malaria hotspots. Source: DHS, 2017-2022; authors' analysis.

The probability of malaria prevalence exceeding the 30% threshold at the 10-km pixel, highlighting areas with a higher likelihood of experiencing substantial malaria transmission across the eight countries, is shown in Figure 7. Overall, while Burkina Faso, Guinea, Ghana, and Liberia had extensive areas unlikely to exceed the 30% threshold, Benin, Cote d'Ivoire, and Togo exhibited larger regions where malaria prevalence remains persistently above critical levels, highlighting areas where intensified malaria control efforts are needed. For instance, in Benin, the highest exceedance probabilities were concentrated predominantly in the southern regions, whereas in Cote d'Ivoire, central and western parts of the country showed the highest risk. Similarly, Nigeria displayed a heterogeneous pattern, with most areas showing low probabilities, but with localised pockets, particularly in the central and southern zones, where the likelihood of exceeding 30% prevalence was higher (>40%). In Togo, exceedance probabilities were moderate across the entire country, with many pixels showing probabilities between 40% and 50%, indicating a consistent, widespread moderate malaria risk.

## Discussion

### Key findings

This study provides a comprehensive, multi-country assessment of malaria among children under five years of age across eight West African countries using microscopy-based data from the Demographic and Health Surveys (DHS) combined with geostatistical modelling techniques. While previous studies have typically focused on single-country analyses or relied on modelled prevalence estimates, our approach integrates nationally representative, laboratory-confirmed infection data with spatial prediction models to produce fine-scale maps of malaria prevalence and exceedance probability across the region. This represents one of the few efforts to apply a consistent geostatistical framework to multiple DHS datasets in West Africa, enabling direct cross-country comparison and identification of transboundary malaria hotspots. By linking individual-level demographic and behavioural factors with spatially explicit risk patterns, the study offers both epidemiological and geographic insights into malaria heterogeneity that are directly relevant for targeting interventions.

A consistent finding across the study region was the significant association between the use of insecticide-treated bednets and lower odds of malaria infection. In Burkina Faso, Benin, Cote d'Ivoire, and Ghana, children who reportedly slept under treated nets were less likely to test positive for malaria. This finding reinforces existing evidence supporting the protective role of long-lasting insecticidal nets (LLINs) in reducing malaria risk among young children [29,30]. LLINs act both as physical barriers and by reducing mosquito survival, thereby contributing to community-level protection when coverage is high. However, their effectiveness depends on sustained and proper use, which can be affected by behavioural and contextual factors such as heat, perceived mosquito density, or net damage [31,32]. These results highlight the need for strategies that go beyond distribution to promote consistent and equitable net utilisation through community engagement and health education.

Another robust pattern identified across all countries was the inverse association between child age and malaria infection. Children under two years of age consistently exhibited lower odds of infection compared with older children. This age-related pattern may reflect both biological and behavioural mechanisms. Infants and younger toddlers are often more closely supervised and sleep indoors under protective nets, whereas older children may be more exposed to mosquito bites through outdoor play and less consistent use of protective measures [31,32]. These findings align with previous multicountry analyses in sub-Saharan Africa showing a gradual increase in malaria infection prevalence with age [33]. They underscore the importance of sustaining malaria prevention measures for older toddlers through age-tailored interventions such as perennial malaria chemoprevention (PMC) and reinforcement of bednet use beyond infancy.

### Comparison with previous studies

Beyond individual-level correlates, the analysis revealed pronounced spatial heterogeneity in malaria prevalence across and within countries, consistent with earlier spatial modelling studies conducted in West Africa [34-36]. These spatial patterns are likely attributable to the interplay of ecological, climatic, and programmatic factors that together shape malaria transmission intensity. Environmental conditions such as rainfall, vegetation density, elevation, and proximity to wetlands are known to create suitable habitats for mosquito breeding and parasite survival [34]. For instance, in Burkina Faso, Millogo *et al.* [34] found that more than 60% of spatial variation in malaria prevalence could be statistically explained by environmental variables including temperature, rainfall, and soil characteristics. Likewise, entomological investigations in southern Benin reported village-level differences in mosquito density associated with proximity to water bodies and vegetation type [35]. In Nigeria, spatial-temporal models have identified clusters of high malaria prevalence linked to rainfall, vegetation indices, and water bodies in the southern and central ecological zones [36]. These consistent patterns across studies emphasise that malaria prevalence in West Africa exhibits strong spatial clustering driven by environmental and infrastructural conditions rather than random variation.

The exceedance probability maps also identified persistent malaria hotspots in southern Benin, central Cote d'Ivoire, and parts of Nigeria, whereas Burkina Faso, Guinea, Ghana, and Liberia showed lower predicted prevalence. The persistence of these high-risk zones may reflect a combination of ecological suitability and incomplete coverage of control interventions. These findings further demonstrate the value of geostatistical approaches in identifying subnational areas where intensified surveillance and intervention could yield substantial impact.

### Policy implications

The findings of this study have direct implications for malaria control and elimination strategies in West Africa. The spatial maps and exceedance probability layers derived from our geostatistical models can serve as practical tools for National Malaria Control Programs (NMCPs) to strengthen data-driven decision-making. Integrating these geospatial outputs into NMCP dashboards would allow health authorities to visualize malaria prevalence and transmission intensity at subnational levels, identify high-burden districts, and allocate resources more efficiently. Such integration aligns with the WHO's call for evidence-based stratification of malaria risk and can complement routine surveillance data to improve operational planning and intervention targeting.

Beyond national applications, the cross-border dimension of malaria transmission underscores the importance of regional collaboration. Several highrisk zones identified in this study such as those spanning southern Benin, western Nigeria, and southern Togo extend across national borders, reflecting shared ecological conditions and population movement patterns. Collaborative planning and synchronised interventions across these borders could help mitigate reinfection risks and enhance the overall effectiveness of malaria control strategies. Regional organisations such as the West African Health Organization (WAHO) and ECOWAS could play a coordinating role by facilitating data sharing, harmonising monitoring tools, and promoting joint response initiatives among neighbouring countries.

In addition to improving surveillance, the geostatistical approach demonstrated here can support targeted intervention delivery. By combining model-based prevalence predictions with exceedance probabilities, NMCPs can identify communities most likely to exceed operational thresholds (e.g., 30% prevalence) and prioritise them for interventions such as indoor residual spraying, seasonal malaria chemoprevention, or enhanced vector control campaigns. Integrating these tools with existing digital health platforms, including malaria information systems and DHIS2 dashboards, would enable more agile and responsive programme management.

Finally, the findings highlight the continued relevance of long-lasting insecticidal nets (LLINs) and community education for sustaining malaria prevention efforts. However, to maximise their impact, control programmes should shift from broad, population-wide approaches toward spatially-targeted and behaviourally adaptive strategies, guided by real-time mapping and subnational risk profiling. Embedding geospatial analytics into NMCP operations can transform malaria surveillance from reactive monitoring to proactive, precision public health planning, ultimately accelerating progress toward malaria elimination in West Africa.

### Limitations and future directions

This study's cross-sectional design limits causal inference, and the associations reported should therefore be interpreted as correlations rather than causal relationships. Incomplete geographic coverage due to insecurity and logistical constraints led to missing data from certain regions, which may have contributed to underestimation of malaria prevalence in some countries. Self-reported variables, such as bednet use, may also be subject to recall and social desirability bias, potentially affecting the accuracy of exposure estimates.

An additional limitation concerns the temporal inconsistency of the Demographic and Health Surveys (DHS) used in this analysis. The surveys were conducted between 2017 and 2022 and thus represent different malaria transmission periods across countries. These time differences may influence cross-country comparability and could partially explain some of the observed spatial heterogeneity. While the geostatistical models were applied consistently across all datasets, future work using temporally harmonised or longitudinal data would provide more accurate insights into dynamic transmission patterns.

Moreover, this study did not incorporate environmental and climatic variables such as rainfall, temperature, or vegetation indices into the spatial models. Their absence limits the ability to explicitly quantify ecological determinants of malaria transmission, even though environmental factors are known to strongly influence vector abundance and parasite development. Integrating remotely sensed environmental covariates into future geostatistical frameworks could enhance predictive performance and help disentangle ecological from socioeconomic effects.

Despite these limitations, the combination of nationally representative, microscopy-based DHS data with geostatistical modelling offers a robust approach for characterising malaria risk in data-limited settings. Future research should expand this framework to include longitudinal data, environmental predictors, and intervention coverage indicators to support more comprehensive, timesensitive malaria surveillance and control planning.

## Conclusions

This multi-country geostatistical analysis provides new insights into the spatial heterogeneity and risk factors of malaria among children under five across eight West African countries. To translate these insights into policy and practice, National Malaria Control Programs should integrate fine-scale geostatistical mapping into their routine surveillance systems to better identify persistent hotspots, monitor progress, and optimize resource allocation.
